# The Potential Revolution of Cancer Treatment with CRISPR Technology

**DOI:** 10.3390/cancers15061813

**Published:** 2023-03-17

**Authors:** Dimitrios Stefanoudakis, Nikhita Kathuria-Prakash, Alexander W. Sun, Melissa Abel, Claire E. Drolen, Camille Ashbaugh, Shiliang Zhang, Gavin Hui, Yeganeh A. Tabatabaei, Yuliya Zektser, Lidia P. Lopez, Allan Pantuck, Alexandra Drakaki

**Affiliations:** 1 School of Medicine, National & Kapodistrian University of Athens, 15772 Athens, Greece; 2 Division of Hematology and Oncology, David Geffen School of Medicine, University of California, Los Angeles, CA 90095, USA; 3 Department of Internal Medicine, Columbia University, New York, NY 10027, USA; 4 Division of Hematology and Oncology, National Cancer Institute, National Institutes of Health, Bethesda, MD 20814, USA; 5 Department of Medicine, David Geffen School of Medicine, University of California, Los Angeles, CA 90095, USA; 6 Department of Urology, David Geffen School of Medicine, University of California, Los Angeles, CA 90095, USA

**Keywords:** CRISPR, CRISPR/Cas9, cancer therapies, novel therapies, cancer prevention, oncology, technology, gene-editing

## Abstract

**Simple Summary:**

Clustered regularly interspaced short palindromic repeats (CRISPR)-CRISPR associated protein (Cas) 9 is a novel technology utilized to modify target genes. Here, we highlight how this versatile technique can be applied to the development of novel therapies for oncology in the preclinical and clinical settings.

**Abstract:**

Immuno-oncology (IO) and targeted therapies, such as small molecule inhibitors, have changed the landscape of cancer treatment and prognosis; however, durable responses have been difficult to achieve due to tumor heterogeneity, development of drug resistance, and adverse effects that limit dosing and prolonged drug use. To improve upon the current medicinal armamentarium, there is an urgent need for new ways to understand, reverse, and treat carcinogenesis. Clustered regularly interspaced short palindromic repeats (CRISPR)-CRISPR-associated protein (Cas) 9 is a powerful and efficient tool for genome editing that has shown significant promise for developing new therapeutics. While CRISPR/Cas9 has been successfully used for pre-clinical cancer research, its use in the clinical setting is still in an early stage of development. The purpose of this review is to describe the CRISPR technology and to provide an overview of its current applications and future potential as cancer therapies.

## 1. Introduction

It has been estimated that over 2 million cancer-related deaths for men and 1 million for women have been avoided between 1975 and 2018. This significant decrease in mortality can be attributed to the efficacy of novel treatments. However, cancer remains the second leading cause of death, behind cardiovascular disease. In the United States, approximately 2 million new cancer cases occurred in 2021. In women aged 40–60 and all individuals aged 60–80, cancer remains the leading cause of death [1].

In recent years, immunotherapeutic regimens have become front line agents in clinical practice. However, one drawback is the difficulty in predicting the efficacy of IO. This difficulty arises from the fact that the response to IO is not linear or directly proportional to the dose of treatment, and there is a lack of reliable biomarkers for predicting response. This current challenge in accurately assessing clinical outcomes suggest a need to establish new criteria and tools to select appropriate patients and to quantify the beneficial effects of such therapies.

Factors that positively affect the efficacy of immunotherapy include programmed death ligand 1 (PD-L1) status, tumor mutation burden, and gene alterations, such as microsatellite instability [2,3,4]. There is no standard methodology to assess PD-L1 positivity given the multiplicity of antibody assays and heterogeneous PD-L1 expression within tumors. While there are efforts to identify and analyze circulating tumor cells, tumor biopsies are currently the standard practice, as both tumor infiltrating lymphocytes (TILs) and the immune profile of the tumor are determinants of clinical outcome [5,6,7,8].

Furthermore, there are challenges identifying biomarkers of durable response among chemotherapeutic agents and targeted therapies, such as inhibitors of the vascular epidermal growth factor (VEGF), mammalian target of rapamycin (mTOR), and cyclin-dependent kinase 4/6 (CDK4/6) [9,10,11]. In addition, finding ways to overcome dose limiting toxicity and predicting toxicity in the future will be beneficial to enhance clinical benefits and prevent early treatment discontinuation [12].

Considering the aforementioned obstacles, in conjunction with the need for personalized treatments, improvements in survival, and treatment tolerability, it is crucial to investigate novel avenues to advance the field of cancer therapeutics. In this direction are various innovative platforms, such as the CRISPR/Cas system. This versatile technology is used as a gene-processing tool to selectively modify DNA sequences at defined sites in the genome with much greater accuracy than conventional genome modification techniques. Current efforts center on identifying clinical applications of this readily available pre-clinical tool yielding promising results.

## 2. CRISPR

As a gene-editing tool, CRISPR rose to prominence in 2012, largely due to its low cost and relative ease of use compared to existing genome editing technologies of the time [13,14,15,16]. The key to CRISPR’s relative simplicity lays in its DNA-targeting mechanism, which uses a strand of RNA that is complementary to the target DNA as a homing beacon for the rest of the CRISPR protein complex. On the other hand, other DNA editing tools, such as zinc-finger nucleases (ZFNs) and transcription activator-like effector nucleases (TALENS), use proteins to target DNA sites of interest [17]. The use of RNA as the target proved to be revolutionary, as designing custom CRISPR guide RNAs is significantly simpler than synthesizing custom ZFN/TALEN proteins.

**Figure 1:**
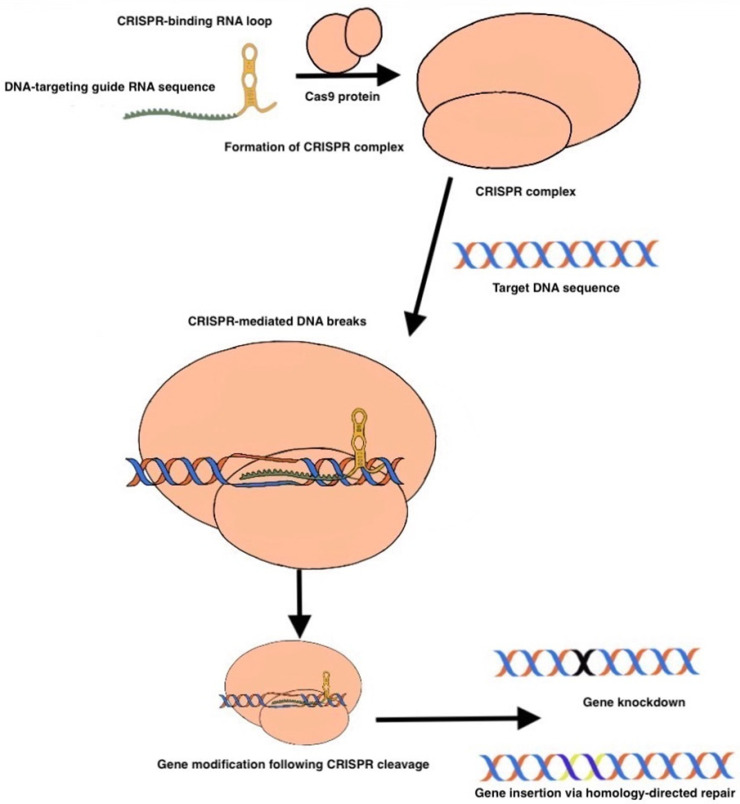
Formation of CRISPR Complex. In the CRISPR/Cas9 system, a guide RNA (gRNA) targeted toward the gene of interest associates with Cas9, forming the CRISPR complex. The gRNA contains a loop region that facilitates Cas9 binding. The CRISPR complex then targets the gene of interest for double strand breakage. After breakage, if a donor template is present, gene knock-in can occur. Otherwise, the DNA sequence will undergo a repair mechanism ultimately leading to a nonfunctional gene (gene knockdown).

CRISPR, as with many biomedical advances, has origins in biology; originally discovered as a bacterial defense mechanism against invading viruses (bacteriophages), diverse CRISPR systems have since been characterized in archaea and phages [18]. Although CRISPR originated as a bacterial defense mechanism against invading viruses (bacteriophages), its potential as a genome editing tool was recently quickly recognized and adapted. At its core, CRISPR consists of two fundamental components: a guide RNA (gRNA) that targets the gene of interest, and a protein complex (called Cas9) that contains a nuclease, which together act as molecular scissors to achieve double-stranded DNA cleavage [19,20]. (Figure 1).

Rough Figure 1 seen above.

**Figure 2:**
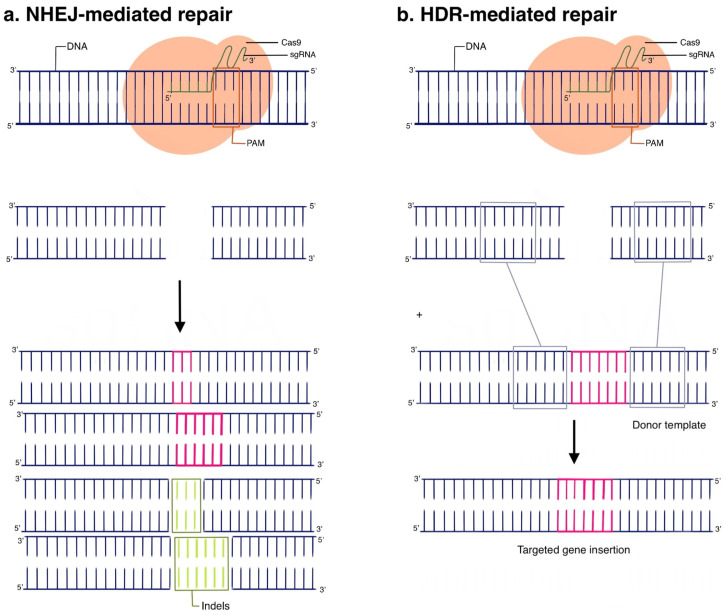
(**a**) NHEJ-mediated repair; (**b**) HDR-mediated repair.

The guide RNA is the linker between the target DNA sequence and the Cas9 endonuclease: gRNA contains a DNA complementarity sequence and a conserved tracrRNA sequence, which is used to bind to Cas9 and thus direct it to the DNA sequence of interest, resulting in cleavage. However, the process of gene editing is not so simple as to conclude with CRISPR-mediated DNA cleavage; our cells are imbued with two primary defense mechanisms against double-stranded DNA damage, known as non-homologous end joining (NHEJ) and homology-directed repair (HDR). Following CRISPR DNA cleavage, human cells typically undergo NHEJ, which by nature of being error-prone, restores the double strand break with insertions and deletions (indels) that result in a nonfunctional gene (Figure 2a). In contrast, HDR exhibits higher fidelity and, in the presence of an appropriate donor sequence, can even introduce new functional genes in place of the cleaved gene. Thus, following CRISPR gene cleavage, the action of NHEJ or HDR-mediated DNA repair ultimately achieves the desired gene-editing effect of either gene-knockdown or complete gene replacement (knock-in) (Figure 2b). Whether the cell undergoes NHEJ or HDR can be biased based on a variety of cell-state factors, and is an area of active research, as gene knock-in via HDR is highly coveted in clinical applications. Given its versatility, CRISPR naturally lends itself to many imaginative applications in cancer diagnostics and treatment, many of which we will highlight.

## 3. CRISPR Preclinical Use

CRISPR technology has been applied in the preclinical space to study its potential in mutation repair, gene editing, oncogene knockdown, and engineered T cell immunotherapy. In an early use case, CRISPR was used to identify a causative mutation in a model of retinitis pigmentosa (RP) by repairing suspect mutations. The “rodless” mouse, which is a preclinical model of RP, has two homozygous mutations that were debated to be the cause of retinal degeneration: a nonsense point mutation and an intronic insertion of a leukemia virus. Wu et al. performed CRISPR-mediated repair to demonstrate that the point mutation is the causative variant of disease. The gene editing was achieved in a stepwise fashion where the first generation animals were mosaic for the corrected allele and second-generation homozygous CRISPR-repaired mice showed rescue and disease amelioration [21]. The ability to use the CRISPR/Cas9 system for gene editing of oncogenic mutations in cell lines and animal models is well-established. With the ability of Cas9 guide RNAs to target specific sequences, numerous creative applications of the CRISPR/Cas9 system can be derived aside from its original knockout activity; one such application involves so-called base-editing, in which deactivated Cas9 lacking its endonuclease activity is mutated into a “nickase” or “nCas9” that can cleave one strand of the target DNA sequence, such that the target is not deactivated completely. Other base editors fuse deactivated Cas9 to enzymes, such as an adenine base editor (ABE) enzyme consisting of a deoxyadenosine deaminase that converts target A-T base pairs to G-C base pairs, resulting in point mutations that modulate target genes [22]. Wen et al. applied CRISPR/Cas9 interference and programmable base editing to telomerase (which includes a reverse transcriptase subunit called TERT) to show that that base-editing of TERT severely compromises cancer cell survival in vitro and in vivo. Specifically, haploinsufficiency of TERT results in telomere attrition and growth retardation in vitro. Thus, inactivating TERT has become a promising means of cancer therapy [22].

Li et al. applied these findings to a glioblastoma model to correct a TERT promoter mutation with a sgRNA and Cas9-fused adenine base editor. This modification blocked the binding of transcription factors to the TERT promoter, reduced TERT transcription and TERT protein expression, and induced cancer-cell senescence and proliferative arrest. Local injection of adeno-associated viruses expressing this sgRNA-guide inhibited the growth of gliomas harboring TERT-promoter mutations [23]. Examples using CRISPR/Cas9 to repair mutations in tumor suppressors and other cancer-associated genes include TP53 in prostate cancer cell lines and *PKC* in colon cancer xenograft models [24,25].

In addition to gene editing, CRISPR/Cas9 has also been used for gene knockdown, a process helpful for identification of novel oncogenes. Using Cas9 fused to green fluorescent protein (GFP), whose fluorescent capability enables tumor localization and quantification, Chen et al. conducted a genome-wide CRISPR loss-of-function screen by transfecting a non-metastatic cancer cell line in mice with Cas9-GFP and then utilizing a genome-scale library of 67,405 sgRNAs. This pool generated metastases when transplanted into immunocompromised mice, and enriched sgRNAs in lung metastases and late-stage primary tumors were found to target a small set of genes. Individual sgRNAs and a small pool of sgRNAs targeting the top scoring genes from the primary screen dramatically accelerated metastasis. This demonstrated CRISPR/Cas9 as a screening method to systematically evaluate gene phenotypes via knockdown in vivo [26]. CRISPR technology has been used to target mutated versions of the *EGFR* gene, the elimination of which has resulted in reduced cell proliferation in vitro and in vivo [27]. More examples include CRISPR-mediated gene knockout in intestinal tumors for functional validation of colorectal cancer driver genes [28] and a CRISPR-screen using a customized pool of sgRNAs in a melanoma cell line which is a novel gene involved in PD-1 resistance [29].

Furthermore, pre-clinical use of CRISPR extends to immunotherapy and T cell engineering. To characterize resistance to PD-1 checkpoint blockade, Manguso et al. applied CRISPR-gene editing to tumors in mice treated with immunotherapy to discover immunotherapy targets, specifically testing over 2000 genes expressed by melanoma cells to identify those that synergize with or cause resistance to checkpoint blockade [29]. Zhang et al. used CRISPR to disrupt CTLA-4 in peripheral CD8+ T cells to show that these cells had enhanced cellular immune response and superior cytotoxicity towards bladder cancer cell lines in vitro [30]. CRISPR technology is even being used in CAR-T cell gene editing to accomplish desired genetic changes [31].

Overall, these preclinical uses of CRISPR demonstrate its vast potential across multiple domains of cancer diagnostics and therapy. The above and more of the following references in this particular article regarding the preclinical applications of CRISPR technology are summarized in Table 1.

## 4. CRISPR in Clinical Practice

Several new gene therapies have received marketing approval by the US FDA and have been made available to patients over the past five years. The first gene therapy to receive FDA approval in the United States was voretigene neparvovec (Luxterna), which was approved to treat retinal dystrophy associated with mutations of the *RPE65* gene [32]. The therapy uses a recombinant adeno-associated virus (AAV) vector to deliver a functioning copy of the *RPE65* gene and was found to effectively improve performance in tests of low light visual perception in a phase 3 clinical trial in children and adults [33]. FDA approval was granted to voretigene neparvovec in December 2017.

Shortly following this success, another AAV-based gene therapy, onasemnogene abeparvovec (Zolgensma), was approved by the FDA for treatment of spinal muscular atrophy (SMA). In the phase 3 clinical trial STR1VE, Zolgensma was found to improve functional outcomes and survival in neonates with infantile onset SMA type 1 [34]. Compared to untreated controls, patients treated with Zolgensma were less likely to require permanent ventilatory support by age 14 months (1/32 vs. 17/23, *p* < 0.0001) and were more likely to achieve functionally independent sitting by age 18 months (14/32 vs. 0/23, *p* < 0.0001). Similar findings were replicated in the European phase 3 trial STR1VE-EU [42]. Efforts to translate the CRISPR/Cas9 system to clinical practice are beginning to meet success, and currently researchers are looking into using the CRISPR/Cas9 technology to modify the survival motor neuron (SMN) gene that is involved in SMA disease.

Many more applications of CRISPR are currently evaluated in clinical trials. A number of these seek to investigate CRISPR for the treatment of hematologic diseases, with sickle cell disease (SCD) and thalassemia being particularly active areas of research. Investigational gene therapies for SCD attempt to repair or replace defective hemoglobin S by providing a modified beta-globin gene or by upregulating fetal hemoglobin F (Hgb F). Lovotibeglogene autotemcel (LentiGlobin BB305) delivers a modified beta-globin gene that encodes an anti-sickling form of hemoglobin, Hb^AT87Q^. Gene therapy with LentiGlobin was first reported in March 2017 in a single-patient case report [43]. A 13-year-old boy with SCD was treated with busulfan conditioning and transplanted with autologous stem cells transduced with the LentiGlobin gene product. Over the 15-month study period, levels of anti-sickling Hb^AT87Q^ increased, reaching 48% of the total hemoglobin concentration by 15 months. The patient experienced no sickle cell crises, and red cell transfusions and pain medications had been discontinued. A follow-up, phase 1–2 study published in 2021 found sustained production of Hb^AT87Q^ up to 18-months after LentiGlobin infusion in 35 patients with SCD. None had episodes of vaso-occlusive pain, acute chest, or stroke following treatment [44]. A phase 3 clinical trial of LentiGlobin in SCD is ongoing at the time of this publication. LentiGlobin has also been studied in patients with transfusion-dependent beta-thalassemia (TDT), and was similarly found to improve clinical outcomes, in this case reducing the need for red blood cell transfusions in 22 patients receiving the therapy [45].

An alternative approach focuses on increasing the production of HgF by targeting BCL11A, a transcription factor that promotes the switch from fetal to adult hemoglobin by repressing HgF. In the CLIMB THAL-111 and CLIMB SCD-111 trials, single patients with TDT and SCD, respectively, were treated with CTX001, a gene silencing therapy directed at *BCL11A*. In both single patient trials, treatment was well tolerated and resulted in a significant increase in HgF concentration and reduction in clinical disease activity [46]. In the following Table 2 are presented summarized the current CRISPR’s applications in clinical practice.

Other applications of gene therapy are under investigation for many other diseases including cystic fibrosis, amyloidosis, Duchenne muscular dystrophy, Huntington’s disease, HIV/AIDS, mitochondrial disorders, and many types of cancer.

## 5. CRISPR in Oncology—Preclinical Use

Proto-oncogenes are regulatory factors in normal developing cells that are capable of controlling and regulating cell differentiation and proliferation processes. However, mutations and modifications to these proto-oncogenes can be very harmful to the normal growing cells by forming oncogenes, initiating cancer formation. On the other hand, there are tumor suppressor genes, which act as the brake pedal against aggressive cell divisions. These tumor suppressors can stop the abnormal growth of the oncogenes when they are in their active form. In healthy human cells, tumor suppressor genes are responsible for the protection of cell cycle overexpression. These tumor suppressors function by monitoring how quickly different cells divide, by repairing the mismatched DNAs, and lastly, by controlling the process of cell death [47]. Some of the most commonly identified and well described tumor suppressor genes are *BRCA1*, *BRCA2*, and *TP53* which are capable of controlling different stages of the cell cycle [49].

The process of tumor development arising from mutations in the DNA is called carcinogenesis, which has four main stages: tumor initiation, tumor promotion, malignant conversion, and tumor progression. Even though a single mutation might not be harmful to the cell, aging leads to an increase in the number of mutations and eventually to carcinogenesis and tumor formation. Another characteristic of cancer cells is genomic instability, specifically chromosomal instability, in which the chromosomal structures change at a high-frequency rate. It is still not yet clear at what stage of cancer initiation this genomic instability starts to become pronounced. These unstable genes have a shorter cell cycle and can bypass the cell division control points that make the cancerous cells begin transforming into malignant cells [50].

Knowing the various causes of carcinogenesis increases the opportunity to discover new techniques to prevent or halt the abnormal cell growth. CRISPR technology and gene-editing tools focus on ways of changing DNA nucleotides to fix the harmful mutations by removing the specific genes and replacing them with the correct ones, with the goal to control and or prevent the process of carcinogenesis [48].

CRISPR/Cas9 has many potential uses in oncology, including creating cancer models, identifying targetable genes, evaluating for resistance mechanisms, and modulating efficacy of immunotherapies [51,52]. As a checkpoint inhibitor immunotherapy and T cell therapies are increasingly approved as first line treatments, optimizing their efficacy is critically important. The CRISPR/Cas9 system’s ability to create site-specific, highly efficient gene knockout makes it a desirable tool to address long-standing challenges, such as T cell exhaustion and tumor microenvironment immunosuppression. Additionally, the gene-editing capabilities of CRISPR/Cas9 offer opportunities to develop a new class of precise, targeted therapeutics in oncology. CRISPR/Cas9 systems have been effective in both completed and ongoing trials in preclinical and clinical phases.

T cell exhaustion is not only a significant problem in endogenous antitumor responses, but significantly limits efficacy of CAR-T cells, especially in immunosuppressive microenvironments [53]. Fraietta et al. analyzed response determinants in relapsed/refractory CLL treated with *CD19*+ chimeric antigen receptor T (CAR-T) cells and demonstrated the presence or absence of T cell exhaustion signatures at apheresis as predictors of clinical outcome [54]. CRISPR/Cas9 can be utilized to eliminate negative regulators of T cell function and persistence in CAR-T cells, theoretically improving clinical response [55].

Another preclinical success in lung cancer is the use of CRISPR technology to target mutated versions of the *EGFR* gene, the elimination of which has resulted in reduced cell proliferation in both in vitro and in vivo [56]. In fact, *EGFRs* were eliminated in a NSCLC cell line that resulted in cancer cell death and tumor size reduction in vivo [57]. Perumal et al. demonstrated that CRISPR technology successfully blocked the tumor suppressor phosphatase and homologous tensin (*PTEN*) in NSCLC, which resulted in increased cancer growth by the promoting Akt pathway [58]. In colorectal cancer associated with *KRAS* or *BRAF* mutations, preclinical studies have identified novel pathways that could eventually be used as clinical targets through genome-wide CRISPR screening in which large libraries of guide RNAs targeted against numerous genes of interest result in large scale knockdown [59,60]. These preclinical advances have paved the way for investigation of the clinical applications of CRISPR/Cas9 in the development of cancer therapeutics.

Since 2018, studies have applied the CRISPR/Cas9 technology to identify potential individualized treatment approaches depending on the subtype of cancer [35]. Various treatment options using different proteins with potential oncogenic effects are currently being studied. Ebright et al. reported that CRISPR/Cas9 can be used as a genome screening tool to identify genes responsible for metastases, such as the overexpression of *RPL15*, a component of the large ribosomal subunit implicated in breast cancer metastasis [61]. Other breast cancer researchers have shown that CRISPR/Cas9 mediated knockout of the *FASN* gene, which is involved in estrogen receptor signaling, can reduce the proliferation and migration of breast cancer cells [36]. While there are no reported applications of CRISPR/Cas9 for triple negative breast cancer (TNBC) thus far, it is established that 80% of *BRCA1* mutations lead to the development of TNBC, and CRISPR/Cas applications can potentially target the poly (ADP-ribose) polymerase 1 (*PARP1*) gene, which is responsible for synthetic lethality in *BRCA1* deficient cells [37]. These are promising advances, suggesting future applications of CRISPR/Cas9 technology for multiple subtypes of breast cancer.

Similarly, in prostate cancer, the estrogen receptor β (*ERβ*) gene was identified and successfully eliminated using CRISPR technology [62]. Although androgen receptor signaling is the main molecular tool regulating growth and function of the prostate gland, *ERβ* is involved in the differentiation of prostatic epithelial cells and numerous antiproliferative actions on prostate cancer cells [62]. While the biological significance of *ERβ* signaling remains unclear, this demonstrates another potential area of application for CRISPR in cancer treatment.

## 6. CRISPR Use in Cancer Prevention

Cancer is fundamentally a genetic disease caused by hereditary and sporadic perturbations in the genome and epigenome that jointly result in dysregulated cell proliferation. As CRISPR/Cas9-based genome editing strategies become increasingly sophisticated, the goal of cancer gene therapy may one day shift from therapeutic intervention to primary prevention. However, before CRISPR can be utilized for cancer prevention in healthy patients, achieving precise and efficient correction of genetic mutations with minimization of off-target effects is essential. Hereditary cancer syndromes associated with known cancer-driving mutations represent a promising therapeutic opportunity for CRISPR/Cas9-assisted mutation repair. As described above, the ability to repurpose the CRISPR/Cas9 system to target and repair tumorigenic mutations in cell lines and animal models is well established. However, despite the success of these genome editing strategies in preclinical studies, there are still many barriers to using CRISPR as an effective cancer prevention strategy in families with hereditary cancer syndromes. Perhaps most notably, hereditary cancer mutations, such as *TP53* in Li-Fraumeni syndrome, mismatch repair genes in hereditary non-polyposis colorectal cancer, and *BRCA1* and *BRCA2* in breast and ovarian cancer syndromes, often affect many different organs and predispose multiple cell types to dysregulated growth [38,39,40]. As a result, achieving effective cancer prevention in these patients would require repairing these mutations systemically and at very high levels of efficiency. This would likely require germline gene editing, which is currently prohibited as the scientific community continues to evaluate the ethical and safety concerns inherent to germline gene therapy.

In lieu of germline editing, CRISPR/Cas9 machinery designed to repair specific hereditary cancer mutations could be delivered systemically or to target tissues having a high likelihood of cancer development. Currently available CRISPR delivery systems that would be amenable to this approach include viral vectors, such as adeno-associated viruses (AAVs), which allow for stable and tissue-selective expression of packaged DNA in transduced cells. However, as somatic cells turn over, repeated delivery of the AAV may be necessary to maintain a high percentage of repaired cells, which introduces potential safety concerns. For instance, persistent Cas9 activity in the host increases the likelihood of off-target DNA cleavage over time, and the host may develop an immune response to the Cas9 protein itself, as certain peptides in Cas9 can act as MHC-binding epitopes [41,63]. Furthermore, it has been shown that CRISPR-edited cells possess fitness defects and are less able to proliferate and differentiate, as CRISPR editing can activate the *p53*-mediated DNA damage response [64].

An alternative, potentially more feasible application for CRISPR/Cas9 in cancer prevention is the targeting of oncogenic viral infections. Before being repurposed for gene editing, CRISPR/Cas9 was identified as a component of the bacterial adaptive immune system that was protective against bacteriophage infection [65]. Accompanied by guide RNAs targeting key genes in the viral genome, the CRISPR/Cas9 system could be employed to promote the clearance of oncogenic viruses, such as HPV, HBV, HCV, and EBV [62]. An important advantage of this approach is the ability to focus on specific cell types (e.g., cervical epithelial cells for HPV, hepatocytes for HBV and HCV) for CRISPR delivery. Furthermore, directing Cas proteins to cleave viral DNA/RNA does not require homology-directed repair, which is significantly less efficient and requires simultaneous delivery of a repair template.

Preclinical work has demonstrated that CRISPR/Cas9 targeting of the HPV *E6* and *E7* genes can reverse the malignant phenotype of cervical cancer cell lines [66]. Similarly, CRISPR/Cas9 has been used to target HBV and HCV in cell and mouse models, which could potentially serve as a strategy for prevention of hepatocellular carcinoma [67,68,69]. Preclinical studies using a Burkitt’s lymphoma B cell line demonstrated that the CRISPR/Cas9 system could also be employed to inhibit EBV replication by using multiple guide RNAs to target different loci in the EBV genome [70,71].

Currently, the most feasible application of CRISPR in cancer prevention may be to target and eradicate oncogenic viral infections, such as HPV in cervical cancer, HBV and HCV in HCC, and EBV in lymphomas and PTLD.

## 7. CRISPR use for Clinical Cancer Treatment

CRISPR/Cas9 has demonstrated promising potential therapeutic applications in oncology, with advances thus far reaching phase I clinical trials [25].

The first phase I trial was conducted by Stadtmauer et al., in which the safety and feasibility of CRISPR-Cas9 to engineer T cells was evaluated [72]. The study enrolled three patients with refractory cancers and used CRISPR/Cas9 to delete two genes that encode endogenous T cell receptor (TCR) genes, reducing TCR mispairing and increasing expression of a cancer-specific TCR transgene. They also removed a gene encoding programmed cell death protein 1 (PD-1) to augment antitumor immunity. All three T cell transfers were successful and persisted for up to nine months, indicating feasibility of the CRISPR/Cas9 system in gene editing for immunotherapies. This novel trial laid the foundation for further investigation in targeted therapies and improving efficacy of immunotherapy.

Two phase I trials have demonstrated the safety and efficacy of CRISPR/Cas9 T cell editing in lung cancer. Lu et al. recruited 22 patients with advanced NSCLC, of whom 12 received treatment with T cells with PD-1 editing by CRISPR/Cas9 [73]. Edited T cells were present in peripheral blood after the infusion, and no severe adverse events were observed. Median progression-free survival was 7.7 weeks and overall survival was 42.6 weeks. The study used next generation sequencing to evaluate for off-target events and median mutation frequency was 0.05%, again demonstrating the safety and feasibility of CRISPR/Cas9 edited T cells. Most recently, Wang et al. recruited 15 patients with mesothelin-positive solid tumors and used CRISPR/Cas9 to generate PD-1 and TCR deficient CAR-T cells specific to mesothelin and evaluated response to dose escalation [74]. Two patients achieved stable disease; circulating edited T cells peaked at days 7–14 and were undetectable after one month, without any severe adverse effects or toxicities, again demonstrating feasibility and safety of CRISPR/Cas9 edited T cells. Liao et al. demonstrated PD-L1 as a possible target for knockout by CRISPR/Cas9 in patients with osteosarcoma [75]. These results are the first steps to establishing the safety and efficacy of CRISPR/Cas9 in the treatment of NSCLC, sarcoma, and likely other malignancies given the significant role of the PD-1/PD-L1 axis in cancer immune escape and therapeutics.

The pre-clinical work of Inturi and Jemth noted above has paved the way for a clinical trial testing the efficacy and safety of CRISPR/Cas9 in targeting HPV *E6*/*E7* to treat persistent HPV and HPV-related cervical intraepithelial neoplasia I [44,76]. In this trial, the Cas9 and guide RNAs are encoded on a plasmid, which is then delivered to cervical epithelial cells via a topical gel that is locally applied to the HPV-infected cervix.

Most recently, Foy et al. developed an approach to knockout two T cell receptor genes using CRISPR/Cas9 technology, and used it to treat 16 patients with different refractory solid cancers in a phase I trial. Each patient received up to three edited TCR products in a dose-escalation clinical trial, which only two patients experiencing cytokine release syndrome or neurotoxicity. Five patients had stable disease as the best response to therapy, demonstrating the feasibility of isolating endogenous T cell receptors and simultaneous knock-out and knock-in technology with CRISPR/Cas9 [77].

Many phase I and II clinical trials are currently ongoing to further investigate the utility of CRISPR/Cas9 technologies in cancer treatments. One long-standing barrier to widespread implementation of T cell therapy has been inherent limitations of autologous donation to manufacture the engineered T cell therapy. Patients with cancer often have suboptimal T cell populations for harvesting, and their malignancy may progress during the in vitro expansion period. Allogeneic T cell immunotherapy remains a highly desirable goal and area of considerable ongoing pre-clinical and clinical research. Currently, there are a number of ongoing phase 1 clinical trials utilizing T cells donated by healthy donors and modified to express CRISPR/Cas9 engineered CAR cells [78,79,80]. Another phase I trial is evaluating autologous T cells engineered to target *CD19* and CRISPR gene edited to eliminate endogenous HPK1 in *CD19*+ leukemia or lymphoma (XYF19 CAR-T cells) [81].

Using CRISPR/Cas9 allows for site-specific, consistent integration, minimizing the risk of heterogenic transgene expression that is more common in typical retro- or lentiviral transduction. CTX110 and CTX112, for example, target *CD19* and is being tested against relapsed or refractory B-cell malignancies [78,82]. Similarly, CTX120 targets the B-cell maturation antigen (BCMA) and is being tested in relapsed or refractory multiple myeloma [79]. Another construct, CTX130, targets *CD70*, and is being tested in advanced, relapsed, or refractory renal cell carcinoma with clear cell differentiation. Many other clinical trials are ongoing, including phase I trials for PD-1 targets in EBV-associated malignancies, as well as phase II trials in *CD19*+ leukemia and lymphoma, relapsed or refractory leukemia and lymphoma, and advanced esophageal cancer [83,84,85,86]. Table 3 summarizes the clinical trials mentioned above.

## 8. CRISPR Use Limitations

As CRISPR/Cas-based genome editing strategies become more efficient, more specific, and more readily deliverable to different cell types, they may one day bring about a new era in the field of cancer prevention and treatment. However, there is still much to learn regarding the long-term safety of CRISPR use in vivo, which will have tremendous impact on our ability to implement it in patients for the primary prevention or treatment of cancer. The applications of CRISPR/Cas9 technology are limited by the potential for off-target activity, which could result in unintended mutations and present a grave risk for the recipient. While studies have demonstrated that this off-target activity is rare, it still represents a major limitation for the widespread usability of CRISPR technology [73]. Other limitations include the potential for immunogenic toxicity from pre-existing antibodies against commonly used bacterial nucleases and the ethical challenges of germ-line gene editing studies [87]. Another consideration revolves around the potential cost of manufacturing and delivering CRISPR/Cas9 based therapies. Finally, but most importantly, the availability of these therapies outside an academic setting may limit the use of this technology as a therapeutic option.

## 9. Conclusions

In conclusion, CRISPR/Cas9 is a novel tool for genome editing that is now established in the preclinical space as an effective strategy for the treatment of cancer and many other diseases. Phase 1 and 2 trials are ongoing to expand its clinical applications. While much work remains to be done before this technology will be implemented widely, CRISPR/Cas9 is an exciting and potentially practice-changing development in the field of immune-oncology and beyond.

## Figures and Tables

**Table 1:** CRISPR’s potential applications.

Target Genes	Cancer Type	Related CRISPR Method	Reference
TERT	Glioblastoma	sgRNA and Cas9-fused adenine base editor	[27]
*TP53*	Prostate cancer	sgRNA and Cas9-fused adenine base editor	[28,29]
*PKC*	Colon cancer	sgRNA and Cas9-fused adenine base editor	[28,29]
Genes on non-metastatic cancer cell line	Lung metastases	Evaluate gene phenotypes via knockdown	[30]
Colorectal cancer driver genes	Intestinal tumors	Evaluate gene phenotypes via knockdown	[32]
Novel gene involved in PD-1 resistance	Melanoma	Evaluate gene phenotypes via knockdown	[33]
CTLA-4	Bladder cancer	Evaluate gene phenotypes via knockdown	[34]
*EGFR*	NSCLC	Blocked the tumor *PTEN* gene	[35]
*KRAS, BRAF*	Colorectal	Genome screening of novel pathways	[36,37]
*RPL15*	Breast cancer metastasis	Genome screening of novel pathways	[38]
*FASN*	Breast cancer	Knockdown	[39]
*PARP1*	Breast cancer	Genome screening of novel pathways	[40]
*ERβ*	Prostate cancer	Genome screening of novel pathways	[41]

**Table 2:** CRISPR in clinical practice.

Status	Name/Trial	Trade Name	Disease	Reference
2017 FDA approved	Voretigene neparvovec	Luxterna	Retinal dystrophy	[43,44]
2019 FDA approved	Onasemnogene abeparvovec	Zolgensma	SMA (Pediatric Patients, < 2 y/o)	[45,46]
Currently evaluated in clinical trials	Lovotibeglogene autotemcel	LentiGlobin BB305	SCD, Thalassemia, TDT	[47]
Currently evaluated in clinical trials	CLIMB THAL-111, CLIMB SCD-111	-	SCD, TDT	[48]

**Table 3:** Current clinical trials.

Clinical Trial	CANCER TYPE	Related CRISPR Method	Reference
Phase 1	Refractory cancers	Delete two genes that encode endogenous TCR and a gene encoding PD-1	[76]
Phase 1	Advanced NSCLC	Edite PD-1 on T cells	[77]
Phase 1	Mesothelin-positive solid tumors	Generate PD-1 and TCR deficient CAR-T cells specific to mesothelin	[78]
Phase 1	Osteosarcoma	PD-L1 possible target for knockout	[79]
Phase 1	Refractory solid cancers	Knockout two T cell receptor genes	[81]
